# Ocular immunology and inflammation under microgravity conditions and the pathogenesis of spaceflight associated neuro-ocular syndrome (SANS)

**DOI:** 10.1038/s41433-024-03005-4

**Published:** 2024-03-05

**Authors:** Mouayad Masalkhi, Joshua Ong, Ethan Waisberg, Andrew G. Lee

**Affiliations:** 1 University College Dublin School of Medicine, Belfield, Dublin, Ireland; 2 Department of Ophthalmology and Visual Sciences, University of Michigan Kellogg Eye Center, Ann Arbor, MI USA; 3 Department of Ophthalmology, University of Cambridge, Cambridge, UK; 4 Center for Space Medicine, Baylor College of Medicine, Houston, TX USA; 5 Department of Ophthalmology, Blanton Eye Institute, Houston Methodist Hospital, Houston, TX USA; 6 The Houston Methodist Research Institute, Houston Methodist Hospital, Houston, TX USA; 7 Departments of Ophthalmology, Neurology, and Neurosurgery, Weill Cornell Medicine, New York, NY USA; 8 Department of Ophthalmology, University of Texas Medical Branch, Galveston, TX USA; 9 University of Texas MD Anderson Cancer Center, Houston, TX USA; 10 Texas A&M College of Medicine, Bryan, TX USA; 11 Department of Ophthalmology, The University of Iowa Hospitals and Clinics, Iowa City, IA USA

**Keywords:** Inflammation, Infection

## Introduction

Spaceflight Associated Neuro-Ocular Syndrome (SANS) refers to a range of neuro-ocular changes experienced by astronauts during long duration space flight (LDSF) missions. The findings in SANS include optic disc edema [1], globe flattening [2], choroidal folds [3], and refractive error shift [4]. Although the exact etiology of SANS is not fully understood it may be multifactorial, involving alterations in cephalad intraorbital, intraocular, and intracranial fluid shifts and secondary changes in tissue and fluid dynamics due to microgravity. Ocular immunology plays a crucial role in maintaining normal eye health by defending against infections. However, spaceflight conditions can disrupt the immune system and may affect the regulation of intracranial, intraorbital, and intraocular pressure. Hence, understanding the relationship between ocular immunology and SANS may be a potential mechanism for developing effective countermeasures to protect astronauts’ ocular health during LDSF.

## Ocular immunology and spaceflight

Crucian et al. [5] explored the dynamics of plasma cytokine levels in astronauts during long-duration spaceflight aboard the International Space Station (ISS). The study collected plasma samples from 28 crewmembers at multiple time points pre-flight, in-flight, and post-flight with a focus on understanding immune system changes and potential health risks associated with space travel [5]. Samples were collected at various intervals, including before, during, and after spaceflight, to capture changes in cytokine levels [5]. It was found while baseline cytokine levels were generally low, spaceflight induced notable increases in certain cytokines, particularly interleukin-8 (IL-8) and TNFα, suggesting a mild inflammatory response [5]. Elevated levels of chemokines such as CCL2, CCL4, and CXCL5 further supported the presence of chronic inflammation during spaceflight [5]. Interestingly, plasma levels of IL-1ra, an inhibitor of the proinflammatory effects of IL-1, were consistently elevated during spaceflight. This increase may represent an adaptive physiological response to inflammatory stress [5]. The study highlights the importance of monitoring cytokine levels as biomarkers for assessing immune system health and the potential health risks associated with long-duration space missions [5].

Some studies have suggested that SANS may be associated with ocular immune alterations (e.g., increase in cytokine production and ocular inflammation) [6]. Additionally, SANS has been linked to changes in ocular blood flow and fluid dynamics, which can lead to optic disc edema and visual impairment [7]. The precise mechanisms underpinning SANS pathophysiology are not fully understood, but they are likely to involve a combination of microgravity-induced changes in fluid dynamics, radiation exposure, and oxidative stress [8–11].

The ocular immune system is responsible for maintaining ocular homeostasis and protecting the eye from infections [12]. Spaceflight-induced immune system alterations include an increase in pro-inflammatory cytokines and a decrease in anti-inflammatory cytokines [6]. One study analyzed blood samples from astronauts who experienced SANS during spaceflight and found alterations in serum cytokine profiles [5]. Specifically, increased levels of the pro-inflammatory cytokine interleukin-6 (IL-6) and decreased levels of the anti-inflammatory cytokine interleukin-10 (IL-10) were observed in astronauts with SANS compared to those without SANS [5].

Microgravity-induced changes can lead to a decrease in the number of lymphocytes and monocytes, thereby impairing immune function [12]. Studies have demonstrated that spaceflight diminishes cytokine production, rendering astronauts more susceptible to infections [13]. This was highlighted in a study in Rooney et al. [14] which identified a potential link between spaceflight-induced stress and immune dysregulation, and contributed to the reactivation of latent herpes viruses in astronauts. The study found increased shedding of these viruses in body fluids during and after missions, highlighting the potential health risks associated with viral reactivation upon return to Earth [14]. Interestingly, it was found that stress-induced immune dysregulation in space may contribute to this viral reactivation [14]. Table 1 lists the prior studies on systemic and ocular inflammation in SANS.

**Table 1:** Summary of studies on the relationship between Systemic and Ocular Immunology and Inflammation with SANS.

Study	Methods	Key findings	Reference
Spaceflight associated neuro-ocular syndrome (SANS) and the neuro-ophthalmologic effects of microgravity: a review and an update	Review Article	Elevated intracranial pressure (ICP) that diminishes upon reentry into the terrestrial 1G environment may engender additional regional structural alterations potentially leading to subsequent inflammation or oxidative stress.	[7]
Spaceflight Effects and Molecular Responses in the Mouse Eye: Preliminary Observations After Shuttle Mission STS-133	Research Article	Spaceflight induces ocular changes in mice, including increased retinal 8-OHdG and caspase-3 levels upon return but decreased at day 7, along with β-amyloid presence in optic nerve fibers. Gene expression shows upregulation of oxidative and cellular stress response pathways upon landing, suggesting reversible molecular damage and induced protective mechanisms in response to spaceflight.	[15]
Plasma Cytokine Concentrations Indicate That In Vivo Hormonal Regulation of Immunity Is Altered During Long-Duration Spaceflight	Research Article	Baseline cytokine levels were generally low, while IL-1ra and chemokines were present. In-flight, IL-8, TNFα, CCL2, CCL4, and CXCL5 increased, suggesting inflammation. Consistent elevations in IL-1ra imply an adaptive response, while post-flight samples trended towards baseline, indicating potential recovery.	[5]
Alterations in adaptive immunity persist during long-duration spaceflight	Research Article	Astronauts experienced persistent immune system alterations including leukocyte redistribution, CD8+ T-cell maturation changes, and reduced T-cell function, as well as altered cytokine production profiles.	[16]

## Conclusion

Overall, the intricate interplay between ocular immunology and the space environment underscores the complex pathophysiology of SANS. The observed increase in pro-inflammatory cytokines coupled with a decrease in anti-inflammatory cytokines suggests a dysregulation of the ocular immune system in spaceflight conditions. These alterations may contribute to optic disc edema, visual impairment, and possibly increased susceptibility to infections. Future studies could explore the specific mechanisms underlying immune dysregulation in microgravity environments, including the role of stress and its impact on viral reactivation. Additionally, longitudinal studies tracking immune parameters before, during, and after space missions may provide valuable insights into the dynamic changes occurring in the ocular immune system.
